# VviWRKY40, a WRKY Transcription Factor, Regulates Glycosylated Monoterpenoid Production by *VviGT14* in Grape Berry

**DOI:** 10.3390/genes11050485

**Published:** 2020-04-29

**Authors:** Xiangyi Li, Lei He, Xiaohui An, Keji Yu, Nan Meng, Changqing Duan, Qiu-Hong Pan

**Affiliations:** 1 College of Food Science and Nutritional Engineering, China Agricultural University, Beijing 100083, China; vale_li@126.com (X.L.); helei@cau.edu.cn (L.H.); anxiaohui@cau.edu.cn (X.A.); yukeji@cau.edu.cn (K.Y.); mn0307@sina.cn (N.M.); chqduan@cau.edu.cn (C.D.); 2 Key Laboratory of Viticulture and Enology, Ministry of Agriculture and Rural Affairs, Beijing 100083, China

**Keywords:** grape (*Vitis vinifera* L.), glycosylated monoterpenoids, WRKY40, transcriptional regulation, abscisic acid

## Abstract

Glycosylated volatile precursors are important, particularly in wine grape berries, as they contribute to the final aroma in wines by releasing volatile aglycones during yeast fermentation and wine storage. Previous study demonstrated that VviGT14 was functioned as a critical monoterpene glucosyltransferase in grape berry, while the transcriptional regulation mechanism of *VviGT14* was still unknown. Here we identified VviWRKY40 as a binding factor of *VviGT14* promoter by both DNA pull-down and yeast one-hybrid screening, followed by a series of in vitro verification. *VviWRKY40* expression pattern negatively correlated with that of *VviGT14* in grape berries. And the suppressor role of VviWRKY40 was further confirmed by using the dual luciferase assay with Arabidopsis protoplast and grape cell suspension system. Furthermore, the grape suspension cell ABA treatment study showed that ABA downregulated *VviWRKY40* transcript level but promoted that of *VviGT14*, indicating that VviWRKY40 was at the downstream of ABA signal transduction network to regulate monoterpenoid glycosylation. These data extend our knowledge of transcriptional regulation of *VviGT14*, and provide new targets for grape breeding to alter monoterpenoid composition.

## 1. Introduction

Aroma is one of the important indicators of grape and wine quality, which directly affects consumers’ acceptance. There are mainly six groups of aroma compounds in *Vitis vinifera* L. wine grapes, namely, terpenoids, norisoprenoids, aromatic compounds, aliphatic volatile compounds, methoxypyrazines, and organo-sulfur compounds. Among these compounds, grape-derived terpenoids have garnered interest because they have a low sensory threshold values and impart pleasant floral/fruity odor. Monoterpenoids are typical volatile compounds of white wines made from aromatic Muscat grape varieties, in which their concentration may exceed the threshold by 100-fold [1]. This group of volatile compounds also detected at supra-threshold concentrations in aromatic non-Muscat varieties such as *V. vinifera* “Riesling” and “Traminer”, but at sub-threshold concentrations in neutral varieties including *V. vinifera* “Cabernet Sauvignon”, “Merlot”, “Chardonnay”, and “Sauvignon Blanc” [2]. Monoterpenoids exists free form of volatile compounds in grapes, but mainly be found in the glycosylated form, which is odorless and non-volatile (up to 95% of the total). However, glycosylated monoterpenoids can be hydrolyzed to release volatile aglycones through acid- and/or enzyme-catalyzed reactions during fermentation and storage of wine [3]. So, the glycoside pool can provide winemakers with tools to manipulate wine aroma because relative enzyme activity and substrate specificity vary among strains and fermentation conditions [4]. Actually, the yeast suppliers also advertise yeast strains with glycosidase activity appropriate for a particular wine style. Moreover, even in finished wine, a latent pool of grape-derived aroma compounds still remains in the glycosylated form that can continue to transform under abiotic conditions [5] Accordingly, we think that grape-derived monoterpenyl glycosides are potential and important aroma precursors. 

Isopentenyl diphosphate (IPP) and its isomer dimethylallyl diphosphate (DMAPP) are the main precursors of terpenoids (Figure S1) [6]. In plants, IPP and DMAPP could be synthesized in two independent pathways: The mevalonic acid (MVA) pathways in cell cytoplast and 2-methyl-D-erythritol-4-phosphate phosphate (MEP) in plastid [7,8]. It is commonly known that monoterpenoids made the greatest contribute to both Muscat and aromatic non-Muscat wines’ aroma, and they are generated from the plastidial MEP pathway. 1-Deoxy-*D*-xylulose 5-phosphate synthase (DXS) is the starting enzyme of this pathway, catalyzing the condensation of glyceraldehyde-3-phosphate and pyruvate into 1-deoxy-D-xylulose 5-phosphate (DXP), followed by converting into geranyl pyrophosphate (GPP, C10) through a series of enzymatic reactions. Finally, the GPP is transformed successively into monoterpenoids and monoterpenol glycosides by terpene synthases (TPSs) and monoterpenol *β*-D-glucosyltransferases (UGTs), respectively. Both TPSs and UGTs are large protein families. Glycosides are formed by the action of glycosyltransferases (GTs), which are a ubiquitous group of enzymes that catalyze the transfer of a sugar moiety from an activated sugar donor. In plant secondary product, the UDP-Glc are typically used as sugar donor, these β-D-glucosyltransferases (UGTs) are belong to the GT1 family of the classification [9,10]. It is predicted that 69 putatively functional *VviTPSs* in *V. vinifera* genome, whereas 43 full-length *VviTPS*s have been functionally identified and each TPS corresponds to several terpenoid products [11,12,13,14]. In contrast, only four monoterpenol *β*-D-glucosyltransferases, VviGT7, VviGT14, VviGT15, and VviGT16, have been characterized biochemically [2,15]. Among them, VviGT7 is mainly responsible for the conversion of neryl and geranyl into their glycosidically bound forms [2], whereas VviGT14 can glycosylate geraniol, *R*, *S*-citronellol, and nerol with similar efficiency and VviGT15 prefers geraniol over nerol [15]. It is also found that VviGT16, another uridine diphosphate glycosyltransferase (UGT), was involved in the biosynthesis of glycosylate monoterpenols and some short-chained and aromatic alcohols with low efficiency [2,14]. Previously, we elucidated that glycosylated monoterpenoids fluctuates but generally increases along with grape berry ripening, and exceeds the concentration of free monoterpenoids in ripening grape berries [16]. Furthermore, we found that the accumulation of glycosylated monoterpenes in *V. vinifera* L. “Muscat blanc à Petit grain” and “Gewurztraminer” varieties was consistent with the expression of *VviGT7* (XM_002276510.2) and *VviGT14* (XM_002285734.2); but *VviGT7* in “Muscat blanc à. Petit grain” grapes is enzymatically inactive because a nucleotide mutation of the enzyme active site occurs in one *VviGT7* allele and a nucleotide insertion appears in another *VviGT7* allele [16]. Abundant *VviGT14* expression is closely positively correlated with the differential accumulation of monoterpenyl glycosides in *V. vinifera* “Muscat blanc à Petit grain” between the two wine-producing regions with distinct climates. In contrast, terpenoid products show a weak correlation with the expression of most *VviTPS* genes [17]. These results demonstrate that understanding *VviGT14* transcription regulation is of importance to control the production of monoterpenyl glycosides in wine grape berries.

There are several studies on the environmental or viticulture effects on monoterpenoid accumulation and the relevant gene expression profile in grape berries [16,17,18,19,20]. On transcriptional regulation, a limited number of transcription factors (TFs) that involved in the regulation of terpenoids biosynthesis have been identified in a wide range of plant species, such as *Arabidopsis*, *Nicotiana attenuata*, *Catharanthus roseus,* and *Actinidia*. These identified TFs belong to the auxin responsive factor (ARF) [21], MYB [22], WRKY [23], bHLH [24,25,26], and AP2/ERF families [27]. Moreover, they primarily participate in the regulation of sesquiterpenoid biosynthesis. In recent years, a few TFs that regulate monoterpenoid biosynthesis have also been identified in some species, such as AaNAC1/2/3/4 and AcEIL1/2/3/4a in *Actinidia*. The two TFs are demonstrated to positively regulate the expression of *AaTPS1* [28], and consequently increase the accumulation of monoterpenes (myrcene, limonene, and terpinolene). Additionally, MsYABBY5 is verified to negatively regulate the limonene biosynthesis in *Mentha spicata* spearmint [29]. Overexpression of *PbbHLH4* could increase the accumulation of free monoterpenes in *Phalaenopsis* [30]. In grapevine, VviERF6, VviERF3L, VviGATA5L, and VviGT-2L are predicted via gene co-expression network analysis to possibly participate in the regulation of monoterpenoid biosynthesis in *V. vinifera* “Cabernet Sauvignon” and “Muscat blanc à Petit grain” berries [17,31]. However, the TFs regulating the biosynthesis of glycosylated monoterpenoids have not been identified functionally in grapes, and even in other plants. 

In our previous study, we demonstrated that *VviGT14* expression is a critical rate-limiting factor affecting the accumulation of glycosylated monoterpenoids [17]. In this study, we screened and identified the functional TFs that regulate the expression of *VviGT14*, the purpose of which is to elucidate the transcriptional regulation of glycosylated monoterpenoid biosynthesis in grape berry. 

## 2. Methods and Materials 

### 2.1. Plant Materials and ABA Treatments

*V. vinifera* “Muscat blanc à Petit grain” leaves and grape berries were sampled in a vineyard in the Shangzhuang Experimental Station (40°14’N, 119°19’E) affiliated to the China Agricultural University. This experimental station is located in Haidian District, Beijing. The leaves were used to isolate genomic DNA and grape berries were used to determine monoterpenoid compounds and analyze gene expression. The grape berries were collected from 42 to 98 days after flowering at an interval of 2 weeks. Approximately, 300 healthy berries were randomly sampled for each replicate, three biological replicates were collected for this research. The fresh berries were immediately used to examine soluble solid content and pH, and the results are presented in Figure S2. The remaining berry samples were quickly frozen with liquid nitrogen and stored at −80 °C until used.

ABA treatment and dual luciferase assay were performed using Chardonnay cell suspension cultures. For the ABA treatment, ABA powder (6.4 mg) was dissolved in 242 μL of ethyl alcohol to prepare the ABA stock solution (0.1 M), and the stock solution was diluted 10-fold with ddH_2_O to prepare the ABA working solution (0.01 M). Different amounts of ABA working solution were added into the cell suspension system to obtain the final ABA concentrations of 0, 10, 50, and 200 μM, respectively. After incubation for 24 h, the cells were collected through filtration and then used for RNA extraction. For the collection of the cells, the bruckner funnel was connected to the suction bottle through a rubber plug, and then the filter paper was placed on the bruckner funnel. The cells were slowly poured on the filter paper. Approximately 100 mg cells were used for the RNA extraction, and the left cells were moved into a RNase-free tube and stored at −80 °C. At least six biological replicates were performed. The cell suspension system was established from Chardonnay petiole callus. When the suspension cells were grown until log phase, they were filtered and inoculated at a cell density of 10% (*v*/*v*) in liquid grape Cormier medium. The suspension culture was grown at 25 °C in the dark with continuous shaking at 90 rpm [32]. 

The leaves of 7-week-old *N. benthamiana* seedlings were used in the assessment of *VviGT14* promoter activity and *VviWRKY40* overexpression experiment. At least three biological replicates were performed using the *N. benthamiana* seedlings. The leaves of 4-week-old *A. thaliana* seedlings were used to prepare protoplasts. These seedlings were planted in 10-cm-dimeter plastic pots with culture stroma in an illuminated chamber and grown under a 16-h light and 8-h dark photoperiod of 28 °C at approximately 600 µmol/m^2^/s light intensity. At least six biological replicates were performed using the *A. thaliana* seedlings. All the experiments were repeated at least three times.

### 2.2. Physicochemical Analysis

For each grape sample, 50 de-seeded berries were pressed and centrifuged to obtain the juice. An electronic pH meter (FE20; Mettler Toledo, Greifensee, Switzerland) was used to measure the pH value of the grape juice and a digital hand-held refractometer (PAL-2, ATAGO, Tokyo, Japan) was used to determine the soluble solid content (SSC, Brix).

### 2.3. Cloning of the VviGT14 Promoter and VviWRKY40 Coding Sequence (CDS)

The genomic DNA of “Muscat blanc à Petit grain” was isolated using the cetyltriethylammnonium bromide (CTAB) method according to the Cold Spring Harbor Protocols [33]. The *VviGT14* (XM_002285734.2) nucleotide sequence was obtained from the Pinot noir (*V. vinifera*) genome on the National Center for Biotechnology Information (NCBI). A 1287-bp length sequence upstream of the translational start site was considered the promoter region of *VviGT14*. Primers (forward: TTGATGGATAATGATGGGTAA, reverse: ATTGATGTGCCCCTGAGC) were designed according to the sequence. The genomic DNA obtained above were used as the template to perform the polymerase chain reaction (PCR) amplification. The PCR conditions were as follows: 94 °C for 10 min, 30 cycles at 94 °C for 30 s, 72 °C for 2 min, and 72 °C for 5 min.

The CDS of *VviWRKY40* was isolated from the berries of “Muscat blanc à Petit grain”. A pair of primers (forward: ATGGAATTCGAATTTATTGATACTTCTC, reverse: TCACCATTTTTCTATCTGAGTTTGGT) was designed using primer 5.0 based on the nucleotide sequence of *VviWRKY40* (XM_010649972.2) from the NCBI [17]. The primers for the cloning of other six WRKY TFs were listed in the Table S1.

Both the PCR products were inserted into the pMD-19 vector and transformed into *Escherichia coli* DH5α. At least seven positive clones were selected and amplified, and then sequenced by Sangon Biotech (Shanghai, China), using the primers M13-47 and M13-48 (Table S1). 

The bioinformatics analysis of the *VviGT14* promoter was performed using Softberry TSSP software (http://linux1.softberry.com/) and PlantCARE software (http://bioinformatics.psb.ugent.be/webtools/plantcare/html/), respectively. Phylogenetic tree was conducted using the neighbor-joining method with MEGA 5.2 [34].

### 2.4. RNA Extraction and Quantitative Real-Time PCR Analysis

The total RNA was isolated using the Plant RNA Isolation Kit (Sigma, MI, USA). Three grapevine genes encoding GAPDH (CB975242), actin (EC969944), and ubiquitin (EC929411) were used as the internal references. The primer sequences of these genes, together with *VviWRKY40*, *VviGT14*, *NbGT*, and *NbEF1α* are listed in Table S1. The qRT-PCR was performed using the Ultra SYBR Mixture (SYBR Green I) (CWBIO, Beijing, China) on an ABI7500 qRT-PCR instrument (ABI, MA, USA) according to the manufacturer’s instructions. The relative change in the target gene expression was calculated using 2^−△CT^; where, △C_T_ = C_target_ − C_ref_ and C_ref_ represents geometric mean of three reference gene threshold cycles (C_Ts_). The mean and standard deviation were estimated. Three biological replicates were performed. The qPCR for each biological replicate was performed at least twice. 

### 2.5. DNA Pull-Down Assay

The BersinBio^™^ DNA pull-down kit (BersinBio, Guangzhou, China) was used to selectively capture the proteins that can bind to the *VviGT14* promoter. The probe solution (4 μg) was denatured at 90 °C for 2 min and incubated with pre-cooled DNA structure buffer, to form DNA probe. Next, streptavidin magnetic beads (Invitrogen, CA, USA) were co-incubated with the DNA probe at 25 °C for 30 min. Approximately 500 mg of grape berries without seeds were crushed to powder in liquid nitrogen to extract proteins. Grape protein solution, DNA probe–magnetic beads, 500 μL of DNA precipitation buffer, 5 μL of poly(dI-dC), 5 μL of protease inhibitor cocktail, 5 μL of DTT, 9 μL of EDTA, and 4.5 μL of EGTA were thoroughly mixed, and then incubated for 1 h at 4 °C. The proteins that bound to the magnetic beads were eluted with an Elution Buffer 1. The eluted fraction was transferred into a new tube and immediately used for protein mass spectrometry analysis using the Q Exactive mass spectrometer (Thermo Fisher Scientific, MA, USA). An Acclaim PepMap C18 column (3 μm × 75 μm × 150 mm) (Waters, MA, USA) was used for the protein separation. The elution solution was composed of solvent A (0.1% formic acid in water) and solvent B (0.1% formic acid in 80% acetonitrile); the gradient elution program was as follows: of 0–5 min 5% B; 5–45 min 50% B; 45–55 min 90% B; 55–65 min 5% B. The flow rate was 0.3 mL/min. Uniprot Homo sapiens database was used for the data retrieval. The data retrieval conditions were listed: Fixed modifications: Carbamidomethyl (C); Variable modifications: Oxidation (M); Enzyme: Trypsin; Maximum Missed Cleavages: 2; Peptide Mass Tolerance: 20 ppm; Mass values: Monoisotopic; Significance threshold: 0.05. 

### 2.6. Yeast One-Hybrid Assay

The yeast one-hybrid (Y1H) assay was carried out using the The Matchmaker™ Gold Yeast One-Hybrid Library Screening System Kit (Clontech, CA, USA). Based on the w-box core element (TTGAC) in the *VviGT14* promoter, a three-repeat w-box sequence was synthesized and subsequently inserted into the pAbAi vector, which acted as bait. We used two effectors, one was grape berry cDNA library for capturing the proteins that can bind to the three-repeat w-box sequence and another is CDS of *VviWRKY40* to verifying whether VviWRKY40 can interact with the w-box motif of the *VviGT14* promoter. The cDNA library of Cabernet Sauvignon grape berries (pGADT7-cDNA) was previously constructed in our lab [35]. The cDNA library and CDS of *VviWRKY40* were fused into the activation domain (AD) of GAL4 into the pGADT7 vector (AD-cDNA or AD-VviWRKY40). The AD-fusion plasmids were transformed into the yeast strain Y1HGold, following the manufacturer’s instructions of the Matchmaker Gold Yeast One-Hybrid Library Screening System (Clontech, CA, USA). To eliminate the self-activation effect of yeast w-box bait strain, an appropriate concentration of AbA antibiotic was carefully selected, which was measured to be 400 ng/mL. The transformants were further selected and grown on the Synthetic Dropout (SD) medium containing 400 ng/mL AbA. The interaction of the TF and w-box element was reflected by the growth status of the transformed yeast on the selected SD medium without leucine. Using the same procedure described above, we also tested the interaction of other six WRKY TFs with the three-repeat w-box sequence. 

### 2.7. Electrophoretic Mobility Shift Assay 

The CDS of *VviWRKY40* was sub-cloned and fused with 6× His-tag peptide into the pET32a vector. Transformed bacteria were grown in LB liquid medium supplemented with 100 μg/mL carbenicillin and 0.2% glucose to absorbance OD_600_ of 0.6–0.8, The VviWRKY40-His recombinant protein was expressed in *E. coli BL21* (DE3) strain (TIANGEN, Beijing, China) by the induction of 1 mM isopropyl-*β-D*-thiogalactopyranoside (IPTG) at 16 °C for 16 h. After centrifugation at 8000 rpm for 15 min at 4 °C, the bacteria were collected and dissolved with the Lysis Buffer (20 mM Tris-HCl, 500 mM NaCl, pH 7.0). The proteins were released from the bacteria by sonication using an Ultrasonic Processor. The VviWRKY40 protein was purified using a Ni^2+^-chelating chromatography column according to the pET system manual (Figure S3), as expected, the size for the VviWRKY40-6×His was 55 kDa (VviWRKY40-36 kDa and the 6×His-19 kDa). A 41-bp *VviGT14* promoter fragment containing one w-box core element with biotin on 3 prime was synthesized (Sangon Biotech, Beijing, China), and the sequence is presented in Table S2. The electrophoretic mobility shift assay (EMSA) was carried out following the instruction manual of the LightShift Chemiluminescent EMSA Kit (Thermo Fisher Scientific, MA, USA). Binding reactions were carried out using 50 ng of His-tag fusion proteins and 15 ng of each of the digoxigenin-labeled promoter fragments at room temperature for 25 min in accordance with a previously published protocol [36]. 

### 2.8. VviGT14 Promoter Activity Assay 

The *VviGT14* promoter activity was assayed in tobacco leaves. The 1287-bp promoter of *VviGT14* was fused with the luciferase (*LUC*) reporter gene (*proVviGT14::LUC*), and then sub-cloned into the reconstructed binary plasmid pCAMBIA1300-LUC using the InFusion HD Cloning Kit (Clontech, CA, USA). pCAMBIA1300-LUC without any promoter (empty *LUC*) was used as the negative control. These constructs were confirmed by sequencing, and subsequently transformed into *Agrobacterium tumefaciens* strain GV3101. Then the agrobacterium suspensions infiltrations were carried out using young but fully expanded leaves of 7-week-old *Nicotiana benthamiana* plants. Equal amount of each agrobacterium suspensions was infiltrated into either side of the 7-week-old *N. benthamiana* leaf as described in our previous study [36]. Subsequently, the infiltrated plants were shifted to an artificial climatic box in dark for 12 h, and then cultured under 16 h light/8 h dark for 60 h at 25 °C. At least six biological replicates were performed. The LUC activity was determined using the Andor iXon charge-coupled device (CCD) imaging apparatus (Andor Technology, Belfast, UK). 

### 2.9. Transient Overexpression of VviWRKY40 in Tobacco Leaves

Transient transformation of the tobacco leaves was conducted following the method of the promoter activity assay described above. The CDS of *VviWRKY40* was inserted into the pGreen II vector, and the constructs were mobilized into *Agrobacterium tumefaciens* strain GV3101. The resultant strain was injected into the tobacco leaves. The leaves infected by *A. tumefaciens* with the empty pGreen II vector were used as the control. These experimental tobacco plants were grown for 5 d at 25 °C under a 16 h light/8 h dark cycle, and the infiltrated leaves were collected to measure the expression of *VviWRKY 40* and *NbGT* (Niben101Scf02537g08001.1). Three leaves from one plant were mixed together and smashed into powder under liquid nitrogen. The three leaves were used as one biological replicate. Three biological replicates were conducted. The two qPCR reactions per replicate were performed. The expression of *NbEF1α* [37], which is reported to be a suitable reference gene for normalizing the transcripts from *N. benthamiana*, was used as the internal control. The primers used in this experiment are listed in Table S1. 

### 2.10. Subcellular Localization of VviWRKY40

The coding sequence of *VviWRKY40* was cloned as C-terminal fusions in the frame with the green fluorescent protein (*eGFP*) gene into the pEZS-NL transient expression vector, and expressed under the control of the cauliflower mosaic virus (CaMV) *35S* promoter. AtFBI1-mCherry, which was provided by Prof. Dapeng Zhang, Qsinghua University, Beijing, is a known nucleus-localized protein, and it can emit red fluorescence [38]. The fusion construct pEZS-NL-VviWRKY40-GFP and the nucleus marker pEZS-NL-AtFBI1-mCherry were co-bombarded into the protoplasts obtained from *Arabidopsis* leaves using a modified polyethylene glycol method [39]. In this experiment, 20 μL (20 μg) DNA (10 μL pEZS-NL-VviWRKY40-GFP and 10 μLpEZS-NL-VviWRKY40-GFP) was applied instead of 10 μL (10 μg) DNA [39]. Thereafter, the protoplasts were incubated for 16 h at 25 °C in dark. The VviWRKY40 localization pattern was determined according to the enhanced green fluorescent protein (eGFP) fluorescence using the Zeiss LSM780 Confocal Scanning Microscope. The nucleus was visualized by mCherry red fluorescent protein fluorescence. The peak excitation wavelengths of eGFP and mCherry were 488 and 587 nm, respectively. All transient expression assays were repeated at least three times.

### 2.11. Dual Luciferase Assay 

The dual luciferase assay was performed using *Arabidopsis* protoplasts and grapevine cell suspension to test the regulatory effect of TF on target gene expression. The regulatory function of VviWRKY40 was first assessed in grapevine cell suspension. The GAL4 luciferase reporter plasmid carried the *LUC* reporter gene and five copies of the *GAL4* activation domain (AD) upstream of the minimal *CaMV35S* promoter (*proCaMV35S 35S*::*LUC*). Another reporter plasmid containing the *REN* reporter gene driven by the *CaMV35S* promoter (*proCaMV35S::REN*) and *GAL4* AD was used as a normalization control. The effector plasmid was constructed through the insertion of *VviWRKY40* upstream of the *GAL4* DNA-binding domain (BD) in the pBD vector (pBD-VviWRKY40). The empty vector (pBD) was used as the negative control, while the pBD construct fused with the *VP16* activation domain (pBD-VP16) was used as the positive control for testing whether the transient system worked or not. At least six biological replicates were performed.

The dual luciferase assay in Chardonnay cell suspension was conducted following the protocol previously described [40]. Briefly, the gateway cloning technology was used to construct the experimental vectors according to the previously published protocols [41]. The CDS of *VviWRKY40* was inserted into the pENTR vector using the pENTR™/D-TOPO^®^ cloning kit (Thermo Fisher Scientific, MA, USA), sequenced, and then transferred by BP cloning into the destination vector (pART7) using the Gateway BP Clonase II enzyme mix (Thermo Fisher Scientific, MA, USA). pLUC-proVviGT14 was the same as described above. The two plasmids carrying *VviWRKY40* and *pVviGT14* were co-transformed into the Chardonnay cell suspension. Cloning of *VviMYBA2* (BAD18978) into pART7 (pART7-VviMYBA2) was described previously [42] and expression of *VviMYBA2* was used in this work as negative control to examine possible unspecific induction of proVviGT14 by unrelated TF. The dual luciferase assay was also performed in *Arabidopsis* protoplasts using different TF vectors, used in the grapevine cell suspension experiment. The *VviWRKY40* sequence was introduced into the pSAT6 vector (*pSAT6*::*VviWRKY40*), and the promoter sequence of *VviGT14* was inserted into pLUC vector as described above. The co-transformation procedure was the same as that used for the subcellular localization of VviWRKY40. 

All transfection experiments were independently repeated at least six times. The mean ratio of firefly (*Photinus pyralis*) to *Renilla* luciferase is defined as the relative activity of *VviGT14* promoter-driven luciferase. All transient expression assays were repeated at least three times.

### 2.12. Extraction and Determination of Monoterpenoids

The free and glycosidically bound monoterpenoids were extracted as previously reported [43,44]. Briefly, 0.5 g of *d*-gluconic acid lactone was added into grape berry powder to inhibit the glycosidase activity, and the clear juice was obtained via centrifugation at 4 °C. Subsequently, 10 μL internal standard 4-methyl-2-pentanol (stock concentration of 1.008 g/L), together with 1 g of NaCl, was added into 5 mL of grape juice in a 20-mL sample vial. Free volatile compounds were collected following the solid phase micro-extraction (SPME) method. Three biological replicates were performed.

After centrifugation at 10,000 g for 5 min, the supernatant was filtered through a membrane filter (2–5 µm, it depends on the turbidity of the supernatant after centrifugation). The obtained clear solution was used for the analysis of free and glycosidically bound terpenes.

Glycosidically bound volatile compounds were extracted using Cleanert PEP-SPE resins (Bonna-agela Tianjin, China, 200 mg/6 mL) that were washed using 10 mL of methanol and then 10 mL of distilled water. Then, 2 mL of clear juice was loaded onto the pre-conditioned Cleanert PEP-SPE cartridges. The free volatile compounds, sugars, and other polar compounds were eluted with Mili-Q water and dichloromethane. Finally, 20 mL of methanol were used to elute the glycosidically bound volatile precursors. The eluate was concentrated to dryness using a rotary evaporator and re-dissolved in 10 mL of 2 M citrate-phosphate buffer solution (pH 5.0). Subsequently, 100 µL of the AR2000 solution (100 mg/mL in 2 M citrate–phosphate buffer, pH 5.0) was added, and then the sample was sealed and vortexed in an incubator at 40 °C for 16 h to release volatile aglycones. The released aglycones were collected using the SPME method mentioned above.

The volatile compounds obtained above were determined using the Agilent 6890 gas chromatograph (GC) coupled with Agilent 5975C mass spectrometer (MS). The analytic protocol has been published previously [16,43]. The compound separation was carried out on a HP-INNOWAX capillary column (60 m× 0.25 mm× 0.25 µm, J&W Scientific, Folsom, CA, USA). The flow rate of carrier gas, helium, was 1 mL/min. The injector temperature was kept at 250 °C. The splitless mode (0.75 min) was used for injection. The GC temperature program was as follows: Initial temperature 50 °C, held for 1 min and increased by 3 °C /min to 220 °C, held for 5 min. The MSD transfer line heater was set at 250 °C. The temperature of ion source and quadrupole were 250 °C and 150 °C, respectively. The mass detector was operated in the full scan mode (m/z 30–350) with electron ionization (EI) mode at 70 eV [43,44,45]. A synthetic matrix was prepared in distilled water containing 200 g/L glucose and 7 g/L tartaric acid and the pH was adjusted to 3.3 with 5 M NaOH solution [17,43]. 

For the determination of monoterpenes in the tobacco leaves, about 5 g of the resulting leaf powder was suspended in 25 mL of citrate-phosphate buffer solution (0.2 M, pH 5.0), and macerated for 3 days at 4 °C. After centrifugation, the supernatant was collected and filtered through a membrane filter (5 µm). Three biological replicates were performed. The clear solution was then used for the analysis of free-form and glycosylated monoterpenes with the same method described above.

The measurements for each biological replicate were carried out at least three times.

### 2.13. Extraction and Determination of ABA

ABA was extracted as the published methods [46], the grape berries without seeds were smashed into powder under liquid nitrogen, and 50 mg powder was blended with extraction solution (80% methanol, *v*/*v*). After centrifuged at 10,000 g for 20 min, the supernatant was eluted through a Sep-Pak C18 cartridge (Waters, MA, USA) to remove polar compounds. Three biological replicates were performed. The elution was used for the determination of ABA content using an UPLC-HRMS system reported by Cao et al [47]. A poreshell EC-120 (3 μm × 4.6 mm × 100 mm) C18 column (Waters, MA, USA) was used for the compound separation. The elution solution was composed of solvent A (0.1% acetic acid in water) and solvent B (0.05% acetic acid in acetonitrile). The flow rate was set at 0.3 mL/min. The gradient elution program was as follows: 0–6.25 min 10% B; 6.25–7.5 min 40% B; 7.5–10.6 min 90% B; 10.6–13.5 min 10% B.

### 2.14. Accession Numbers

Sequence data of this study can be found in the GenBank, Grape Genomes (http://genomes.cribi.unipd.it/grape/), or Solgenomics database (https://solgenomics.net/organism/Nicotiana_benthamiana/genome) under the following accession numbers: XM_002285734.2 (*VviGT14*), EC969944 (*VviActin*), EC929411 (*VviUbiquitin*), CB975242 (*VviGAPDH*), TC57089 (*VviNECD1*), VIT_204s0008g05760 (*VviWRKY40*), and Niben101Scf02537g08001.1 (*NbGT*).

## 3. Results

### 3.1. Characterization of the VviGT14 Promoter Region

**Figure 1:**
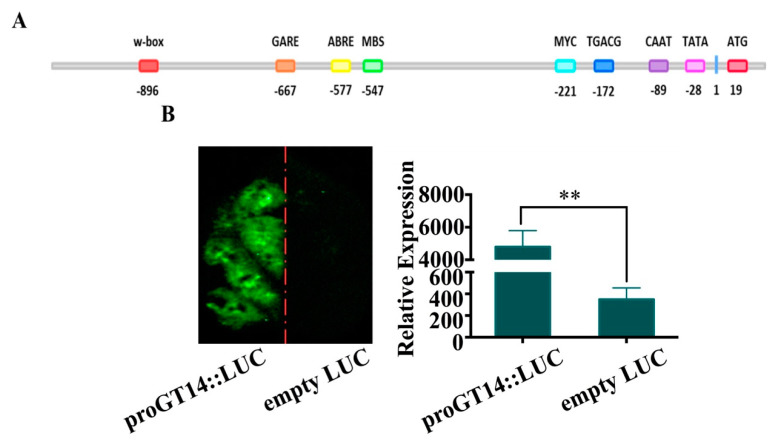
Verification of the *VviGT14* promoter activity in tobacco leaf. **A.** Diagram of main *cis*-acting elements in the *VviGT14* promoter region. **B.** Transient expression of the *VviGT14* promoter-driven luciferase (LUC) gene; tobacco leaves were transformed with equal amount of the proVviGT14::LUC construct (left side of the leaf) and empty LUC construct (right side of the leaf). Right panel: fluorescence imaging. Left panel: relative expression of the *LUC* gene in the experimental and control groups. The data are expressed as the mean ± SD from at least six biological replicates. ANOVA tests (**, *P* < 0.01).

A 1287-bp sequence upstream of the *VviGT14* translation start site (ATG) was obtained. The transcriptional start site (TSS) is positioned at −80 bp, and two typical promoter *cis*-acting elements, CAAT-box and TATA-box, are located at −91 and −34 bp upstream of the TSS (Figure 1A). This sequence presented promoter activity, based on the significantly enhanced fluorescence signal at the side of tobacco leaves where the plasmid carrying *VviGT14* promoter-driven *LUC* gene was infiltrated (Figure 1B). 

Additionally, the *VviGT14* promoter contained several *cis*-acting elements related to the adversity stress and hormone responses, such as an ABA response element (ABRE) at −578 bp, CGTCA-motif at −173 bp, and GARE-motif at −668 bp, which respond to methyl jasmonate and gibberellin, respectively. Three potential TF-binding motifs were found, namely, a w-box element, MBS element, and MYC-binding motif. Among them, the w-box (TTGAC) at −896 bp is predicted to be a WRKY family-binding domain and the MBS (CGGTCA) at −547 bp is a putative MYB family-binding motif (Figure 1A). The result provides a reference for the subsequent research.

### 3.2. Screening of TFs Binding to the VviGT14 Promoter

**Figure 2:**
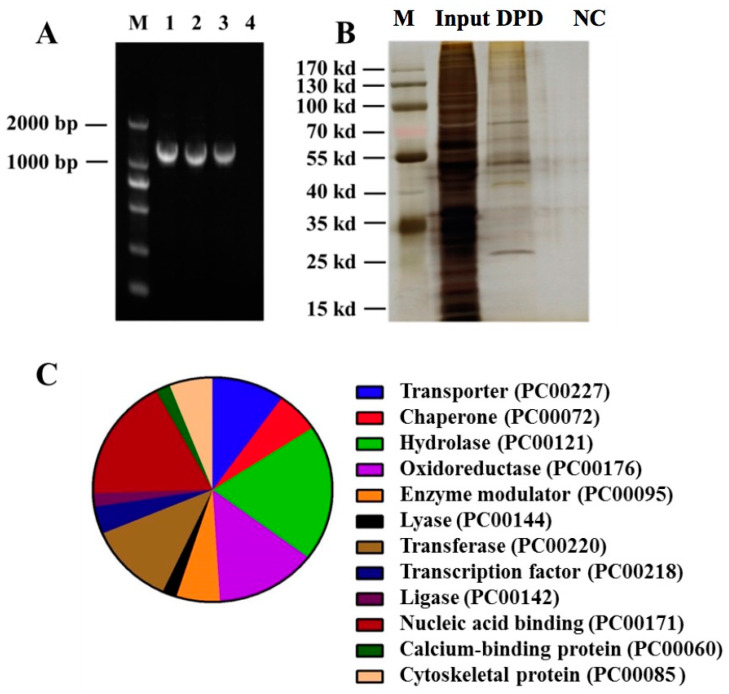
Screening of transcription factors binding to the *VviGT14* promoter in DNA pull down assay. **A.** Agarose gel electrophoresis of the *VviGT14* promoter sequence of 1287-bp length. M represents marker, line 1 represents the *VviGT14* promoter, line 2 represents the *VviGT14* promoter with probe, line 3 represents the remaining of *VviGT14* promoter with probe in the interaction buffer after the incubation of the *VviGT14* promoter with probe and magnetic beads, indicating that the magnetic beads can bind with the extreme promoter on them. Line 4 represents the final elution buffer of magnetic beads, indicating that the *VviGT14* promoter is completely eluted from the magnetic beads. The eluted solution is subsequently used for the mass spectrometry assay. **B.** Polyacrylamide gel electrophoresis silver staining of the *VviGT14* promoter pull-down grape berry proteins. NC represents negative control, comprising loading buffer only. The DPD group represents DNA pull down group, comprising a *VviGT14* promoter-specific pull-down probe. Input represents the positive control, comprising the total proteins obtained from grape berry. **C.** Categories of proteins identified via the pull-down experiment and protein mass spectrometry analysis.

Two strategies were adopted to screen the proteins binding to the *VviGT14* promoter. First, the VviGT14 promoter probe was prepared (Figure 2A). A total of 239 proteins were captured by employing the DNA pull-down method (Figure 2B), among which 10 proteins were predicted to be TFs (Table 1). The Gene Ontology analysis revealed that the nucleic acid-binding proteins accounted for 24.6% of total captured proteins, followed by oxidoreductases (13.7%) and transferase (11.8%). The 3.9% proteins were deduced to be TFs (Figure 2C). Upon score ranking from high to low, the TF VviWRKY40 (VIT_204s0008g05760) was provided attention because of the high score. 

**Figure 3:**
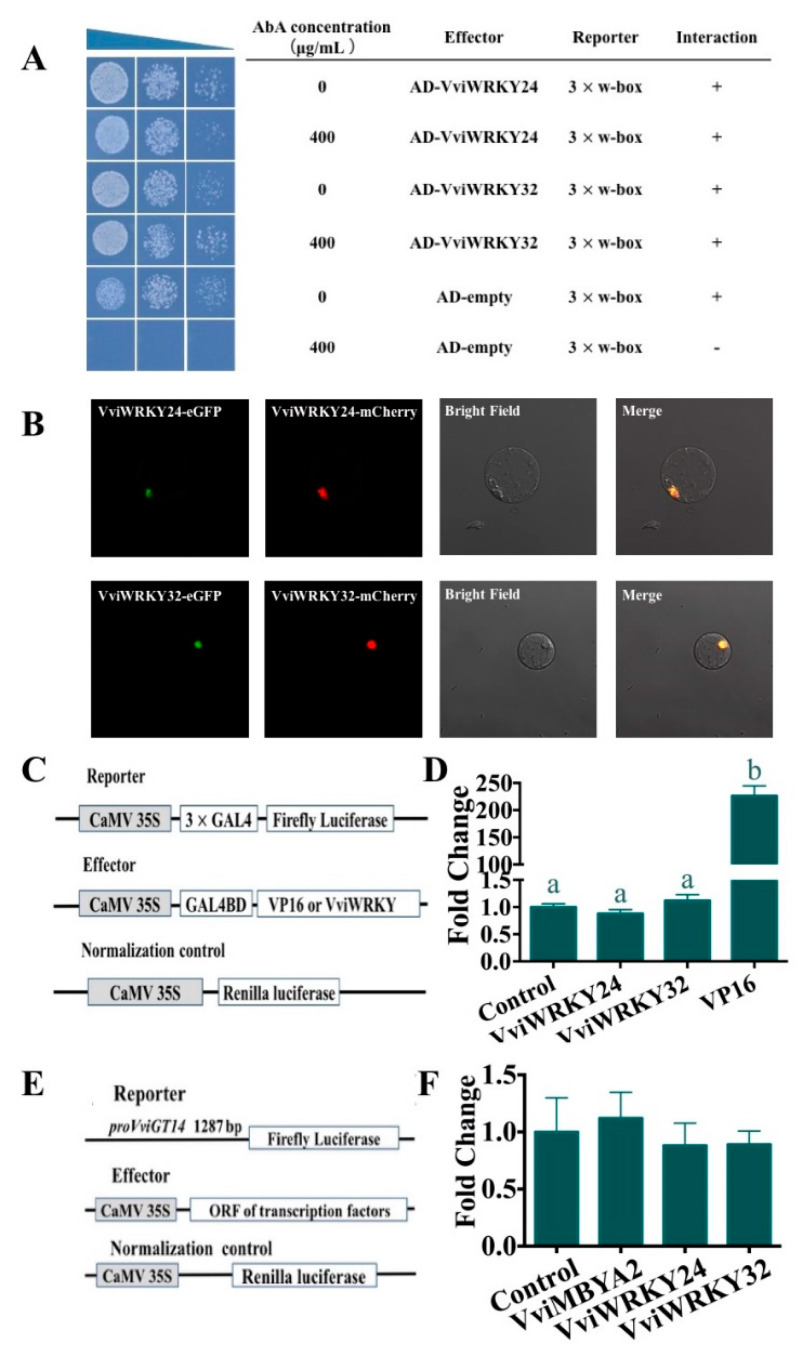
Identification of potential regulation of VviWRKY24 and VviWRKY32 on *VviGT14.* **A**. Yeast-one-hybrid assay showing that yeasts carrying a combination of effector and reporter vectors can grow on the medium without or with 400 ng/mL antibiotic aureobasidin A (AbA), whereas yeasts with AD and 3× w-box cannot grow on the media with 400 ng/mL AbA. The present result indicates that both VviWRK24 and VviWRKY32 can bind with the 3 repeat w-box sequence. **B**. Subcellular localization of VviWRK24 and VviWRKY32 in *Arabidopsis* protoplast. The graphs from left to right represent VviWRKY-eGFP, nucleus marker-mcherry, bright field, and the merge images. The present result indicates that both of the two TFs are specifically located in the cell nucleus. **C.** The reporter, effector and normalized control constructs used in the transcriptional activity assay. **D.** Transcriptional activation domain analysis of VviWRKY24 and VviWRKY32; The transcriptional activation functions of VviWRKY24 and VviWRKY32 were tested using the luciferase assay. The control represents empty pBD vector and its fold change is set as 1. VP16, an unusually potent transcriptional activator, is regarded as a positive control, and its activity change exceeds 200-fold. The error bars represent S.E.s from six biological replicates. Significant differences relative to the control are determined using the one-way ANOVA tests (**, *P* < 0.01). The present result indicates that both of the two TFs lack the transcriptional activation domain. **E.** The reporter, effector and normalized control constructs used in the dual luciferase assay. **F.** The transcriptional regulation assessment of VviWRKY24 and VviWRKY32 on the *VviGT14* promoter activity in a transient expression system in *Vitis vinifera* L. “Chardonnay” cell suspension using dual luciferase assay. VviMYBA2 (BAD18978), a specific transcription factor in anthocyanin biosynthesis, is taken as negative control to test the reliability of this reaction system. The present result indicates that both of the two TFs have no transcriptional regulation effects on *VviGT14* promoter. The data were shown as the mean ± SD from at least six biological replicates. ANOVA tests (*, *P* < 0.05. **, *P* < 0.01).

**Figure 4:**
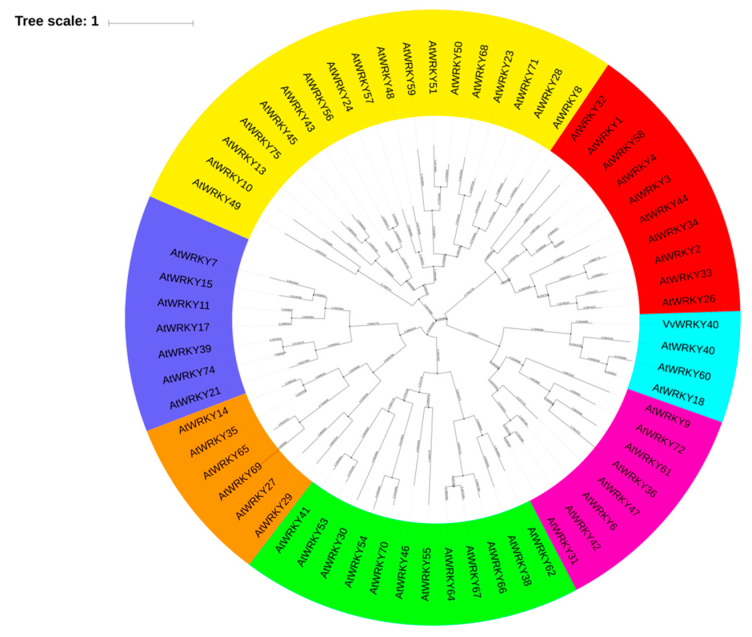
Neighbor-joining phylogenetic tree constructed from the deduced amino acid sequences of VviWRKY40 and other WRKY family transcription factors from Arabidopsis retrieved from the NCBI’s RefSeq database using MEGA 5.20.

Besides, yeast one-hybrid library screening was performed using the three tandem w-box motif repeats as bait, based on the w-box core sequence of the *VviGT14* promoter as described above. To eliminate the effect of yeast self-activation, the minimal inhibitory concentration of aureobasidin A (AbA) was examined for the bait strain harboring pAbA-w-box. It was observed that none of the clones could grow on the SD-Ura medium when the AbA concentration was up to 400 ng/mL (Figure S4). Under this selected concentration, we obtained 32 Y1HGold yeast clones on the Y1H screening media (Figure S5), seven of which were predicted to be transcription factors and they all belong to WRKY family (Table 2). Subsequently, these WRKY TFs were separately cloned and a modified yeast one-hybrid assay was implemented using the pGADT7-VviWRKY vector as an effector and using the pAbAi vector carrying three-repeat w-box sequence as a reporter. The result verified that in addition to VviWRKY40, only VviWRKY24 and VviWRKY32 could bind to the 3x w-box sequence (Figure 3A), and both of them were located in the cell nuclear and lacked transcriptional activation function (Figure 3B,C). But VviWRKY24 and VviWRKY32 showed no significant influence on the *VviGT14* promoter activity in the dual luciferase assay using grapevine suspension cell line (Figure 3D). So we speculated that VviWRKY40 could be an important regulatory factor controlling the expression of *VviGT14*. In this paper, the function of VviWRKY40 was assessed in detail. A phylogenetic tree analysis using the neighbor joining method was applied among VviWRKY40 identified in this study and all the WRKY family transcription factors from Arabidopsis, VviWRKY40 belongs to the IIa subfamily (Figure 4), and this protein is composed of 317 amino acids with a 963-bp CDS (NM_001281019.1).

### 3.3. Validation of VviWRKY40 Interaction with the VviGT14 Promoter

**Figure 5:**
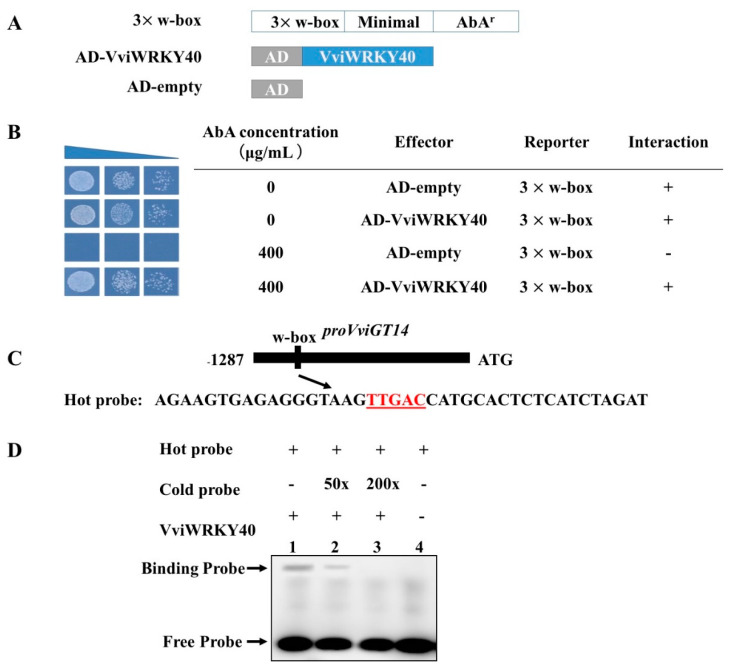
Identification of VviWRKY40 binding to the *VviGT14* promoter using the yeast one hybrid (Y1H) (**B**) and electrophoretic mobility shift assay (EMSA) (**C**). **A.** The coding sequence of VviWRKY40 is introduced into the pGADT7 vector, and the three tandem w-box sequence into the pAbAi vector. **B.** Yeast one hybrid assay showing that yeasts carrying a combination of effector and reporter vectors can grow in the medium without or with 400 ng/mL antibiotic aureobasidin A (AbA), whereas yeasts with AD and 3× w-box could not grow on the media with 400 ng/mL AbA, indicating that the VviWRKY40 can bind with the 3 repeat w-box. **C.** Probes used for the EMSA. The w-box motif on the *VviGT14* promoter is highlighted in red. **D.** EMSA showing that VviWRKY40 binds to the w-box motif of the *VviGT14* promoter. The hot probe is a 3’ biotin-labledfragment of the *VviGT14* promoter, while the cold probe is an unlabeled competitive probe. + and − represent the presence and absence of the VviWRKY40 or the probe, respectively, 50× and 200× indicate 50-fold and 200-fold excess of unlabeled competitive probes, respectively.

Using the pGADT7-VviWRKY40 vector as an effector and using the pAbAi vector carrying three-repeat w-box sequence as a reporter (Figure 5A), the Y1HGold yeast co-transformed with proGT14-AbAi and pGADT7-VviWRKY40 could grow normally on the SD medium containing 400 ng/mL AbA, whereas the yeast carrying proGT14-AbAi and pGADT7 could not grow (Figure 5B), which proved that VviWRKY40 can definitely bind with the repeated w-box sequence of the *VviGT14* promoter. The interaction of VviWRKY40 with the *VviGT14* promoter fragment was further tested using the electrophoretic mobility shift assay (EMSA). In this method, only one w-box was contained in the reporter fragment, which was the same as the *VviGT14* promoter (Figure 5C). Binding of the VviWRKY40 protein to the *VviGT14* promoter fragment was observed when the unlabeled competitive probe (cold probe) was not added or added no more than 50-fold (Figure 5D). The result also supported that VviWRKY40 can bind to the w-box-containing recognition site in the VviGT14 promoter.

### 3.4. VviWRKY40 Localizing in the Cell Nucleus and Lacking Transcriptional Activation Function 

**Figure 6:**
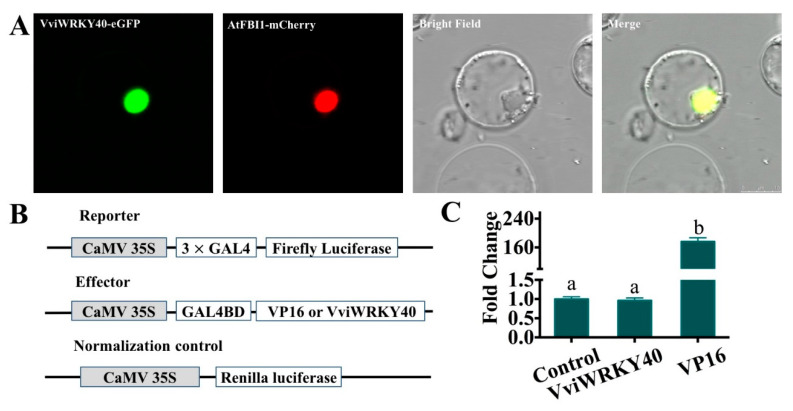
Subcellular localization and transcriptional activity assay of VviWRKY40 in *Arabidopsis* protoplasts. **A.** Subcellular localization of VviWRKY40 in *Arabidopsis* protoplast. The graphs from left to right represent VviWRKY40-eGFP, nucleus marker-mcherry, bright field, and the merge images, bar = 5 μm. **B.** The reporter, effector and normalized control constructs used in the transcriptional activity assay. **C.** Transcriptional activation domain analysis of VviWRKY40. The transcriptional activation function of VviWRKY40 was tested using the luciferase assay. The control represents empty pBD vector and its fold change is set as 1. VP16, an unusually potent transcriptional activator, is regarded as a positive control, and its activity change reaches 160-fold. The error bars represent SEs from at least six biological replicates. Significant differences relative to the control are determined using the one-way ANOVA tests (**, *P* < 0.01).

To understand the subcellular localization of VviWRKY40, the *VviWRKY40–eGFP* and *AtFBI1-mCherry* fusion constructs were co-transfected into *A. thaliana* protoplasts. AtFBI1-mCherry is a known nucleus-localized protein, and it can emit red fluorescence. As expected, it was observed that both green fluorescence and red fluorescence appeared in the nucleus, which indicated that VviWRKY40 functions in the cell nucleus (Figure 6A).

To determine whether VviWRKY40 possesses the transcriptional activation function, the *VviWRKY40* gene was fused into the GAL4 DNA-binding domain (pBD-VviWRKY40) and the luciferase (*LUC*) gene was linked to the GAL4 activation domain (pLUC). Transactivation of the *LUC* gene was examined relative to the *Renilla* luciferase (*REN*) gene under the control of the constitutive cauliflower mosaic virus 35S (*CaMV35S*) promoter (Figure 6B). These constructs were co-expressed in *Arabidopsis* protoplast, and the luciferase activity was quantified. The results indicated that, comparing with the negative control (pBD), the luciferase activity was almost unchanged in the pBD-VviWRKY40 construct; alternatively, the positive control (pB-VP16) showed an approximately 175-fold induction of relative *LUC/REN* expression (Figure 6C). These data revealed that VviWRKY40 lacked the transcriptional activation function, and it may act as a transcription repressor.

### 3.5. Negative Regulation of VviWRKY40 on VviGT14 

**Figure 7:**
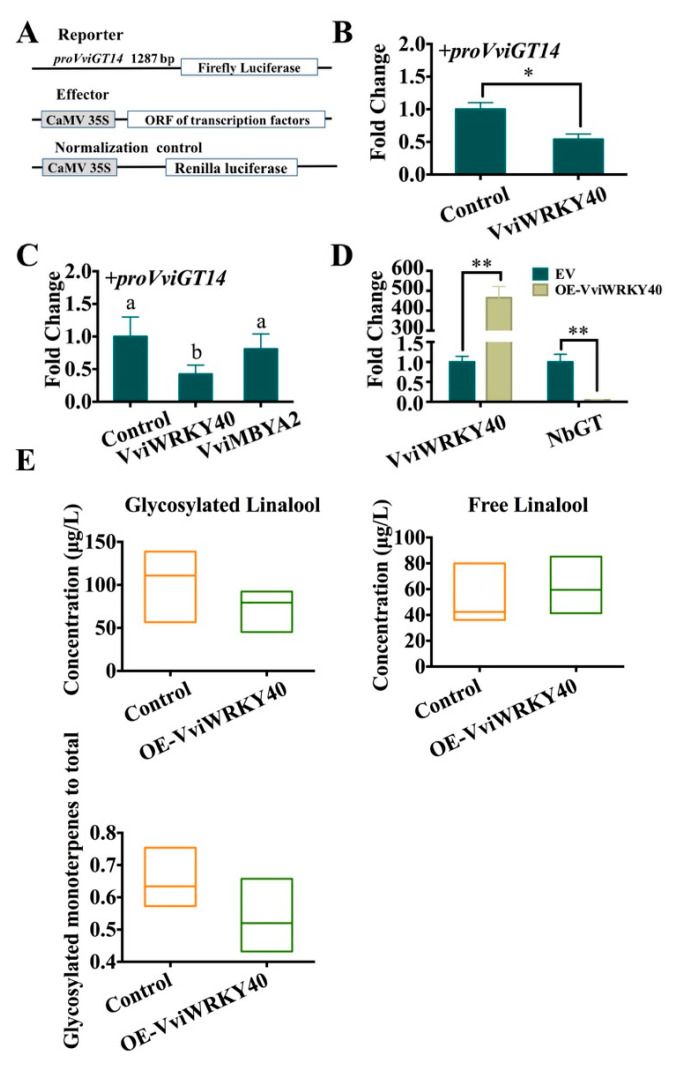
Regulation of VviWRKY40 on the promoter activity of *VviGT14* in a transient expression system (**A**, **B** and **C**) and the expression of the related genes in the VviWRKY40 over-expressed tobacco leaves (**D**). **A**. The reporter, effector and normalized control constructs used in the dual luciferase assay. **B**. The suppression of VviWRKY40 on the *VviGT14* promoter activity in a transient expression system of *Arabidopsis* protoplasts using dual luciferase assay. The control represents pART7 without any transcription factors. The data are displayed as the mean ± SD from at least six biological replicates. ANOVA tests (*, *P* < 0.05). **C.** The suppression of VviWRKY40 on the *VviGT14* promoter activity in a transient expression system in *Vitis vinifera* L. “Chardonnay” cell suspension using dual luciferase assay. VviMYBA2 (BAD18978), a specific transcription factor in anthocyanin biosynthesis, is taken as negative control to test the reliability of this reaction system. The data are expressed as the mean ± SD from at least six biological replicates. **D.** Relative expression of *NbGT* and *VviWRKY40* in transiently over-expressed tobacco leaves, EV, as a control, indicates empty-vector expressed tobacco leaves; OE-VviWRKY40 indicates VviWRKY40 overexpressed tobacco leaves; The data were shown as the mean ± SD from at least three biological replicates. ANOVA tests (*, *P* < 0.05. **, *P* < 0.01). **E.** The boxplots of the concentrations of free-form (left) and glycosylated linalool (medium) in the Vv-WRKY40 transiently overexpressed and control tobacco leaves as well as the ratio of glycosylated linalool to the total (right). At least three data are contained in a boxplot. At least three biological replicates were performed.

In the dual luciferase assay, the *proVviGT14*-driven luciferase reporter and CaMV35S-driven *VviWRKY40* effector were separately constructed and then co-transferred into the protoplasts or cell suspension (Figure 7A). VviWRKY40 was found to suppress the activity of the *VviGT14* promoter with a LUC/REN ratio of less than 0.6 fold in *Arabidopsis* protoplasts, indicating that VviWRKY40 is a suppression effector of *VviGT14* expression (Figure 7B). 

To eliminate the interference of hetero-cellular environment on the regulatory effect of VviWRKY40, the dual-luciferase reporter assay was also performed in homologous grapevine cell suspension system (Figure 7C). We found that VviWRKY40 significantly declined the activity of the *VviGT14* promoter with a LUC/REN ratio of 0.4 fold. In comparison, the anthocyanin biosynthesis specific transcription factor VviMYBA2 (BAD18978), which was used as negative control, did not cause any change of the *VviGT14* promoter activity. VviWRKY40 was demonstrated capable of binding to the promoter of *VviGT14* and to downregulate the transcriptional activity. 

Second, to illustrate the homologous regulation of VviWRKY40 on the *VviGT14* gene expression *in vivo*, *VviWRKY40* was transiently overexpressed in tobacco leaves mainly because it is difficult to obtain a stable transformation system in grapevine. The *NbGT* nucleotide sequence shares approximately 68.0% identity with the *VviGT14* sequence of grapevine. As expected, VviWRKY40 was overexpressed by more than 400-fold in the overexpression tobacco leaves; correspondingly, the expression of *NbGT* was strongly repressed (Figure 7D). In addition, free-form and glycosylated linalool was measured in the transient overexpression and control tobacco leaves. The concentration of free-form linalool in the OE-VviWRKY40 leaves tended to be slightly higher than that in the control. On the contrary, both the concentration of glycosylated linalool and the ratio of glycosylated to total linalool were lower in the OE-VviWRKY40 leaves (Figure 4E). This demonstrates that VviWRKY40 can downregulate the expression of *NbGT* and suppresses the production of glycosylated linalool.

### 3.6. Negative Correlation between VviWRKY40 Expression and VviGT14 Expression during Grape Berry Ripening

**Figure 8:**
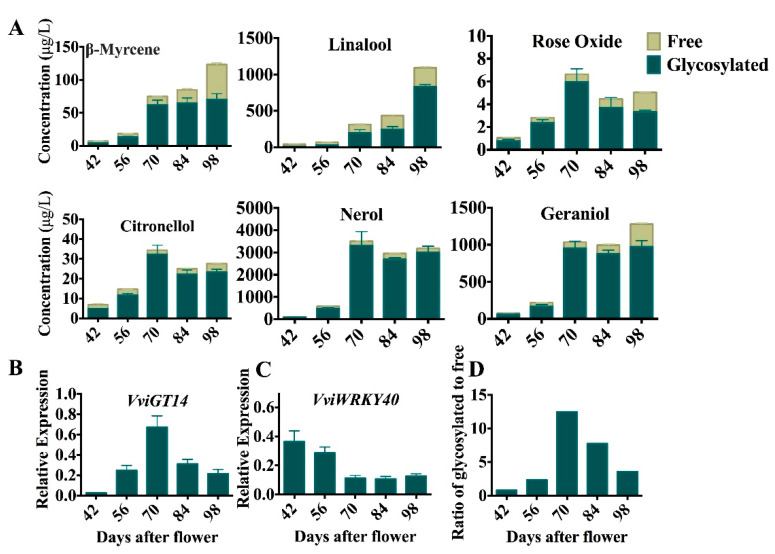
Accumulation of main monoterpenes and the expression of *VviWRKY40* and *VviGT14* during *Vitis vinifera* L. “Muscat blanc à Petit grain” grape berry ripening, mean values and SE were calculated from three independent experiments. The data are expressed as mean ± SD of three replications in **A**, **B** and **C**, **A.** Change in the concentration of main monoterpenes during berry ripening. **B.** Relative expression of *VviWRKY40* during berry ripening. **C.** Relative expression of *VviGT14* during berry ripening. **D.** Ratio of glycosylated monoterpenes to free.

To test whether VviWRKY40 similarly downregulates the expression of *VviGT14* in developing grape berries, we analyzed the expression pattern of the two genes during maturation of “Muscat blanc à Petit grain” berries. As discussed previously [17,48], the concentration of glycosidically bound monoterpene precursors was considerably higher than that free-type monoterpenoids; moreover, the concentrations of free and glycosylated monoterpenes were low at the early developmental stage (42 and 56 days after flowering), and then suddenly peaked at the *véraison* (70 days after flowering) and slightly declined in the following ripening period. Rose oxide, geraniol, *β*-myrcene, citronellol, linalool, and nerol were six monoterpenoid components with the highest concentrations in mature berries of “Muscat blanc à Petit grain”. Among them, linalool, *β*-myrcene, geraniol, and nerol gradually accumulated with berry maturation, whereas rose oxide and citronellol slightly reduced after *véraison* (Figure 8A). As we observed previously [16], the *VviGT14* expression was greatly promoted at 70 days after flowering (Figure 8B), and the *VviWRKY40* expression was declined correspondingly (Figure 8C), which showed a potentially negative correlation between the two genes. Meanwhile, the upregulation of *VviGT14* expression was in good agreement with the rapid accumulation of total glycosylated monoterpenoids, particularly the ratio of total glycosidically bound monoterpenes to free monoterpenes (Figure 8D). Based on the above evidence from in vitro and in vivo experiments, we can reasonably conclude that VviWRKY40 negatively regulates the expression of *VviGT14* by binding with the gene promoter in grape berry.

### 3.7. ABA Downregulates VviWRKY40 and Upregulates VviGT14 in Grapevine Cell Suspension

**Figure 9:**
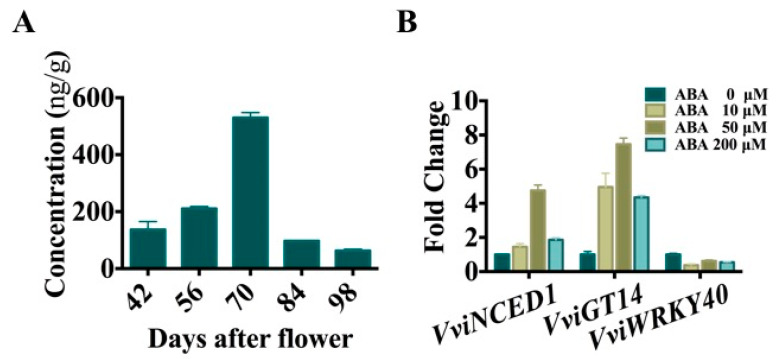
Change of aureobasidin A (AbA) concentration during *Vitis vinifera* L. “Muscat blanc à Petit grain” grape berry ripening (**A**) and the expression of the *VviGT14*, *VviNCED1*, and *VviWRKY40* genes in *Vitis vinifera* L. “Chardonnay” suspension cell system treated with different concentrations of ABA. (**B**). At least three biological replicates were carried out, and ANOVA was performed.

ABA is a commonly known hormone, and its concentration rapidly increased at the *véraison* stage (E-L 35), and then decreased along ripening (Figure 9A), which showed similar trend with the expression of *VviGT14* (Figure 8B). To understand whether ABA associates with the transcriptional regulation of VviWRKY40 on *VviGT14*, we treated grapevine cell suspension with a gradient concentration of ABA. The results showed that ABA application led to more than two-fold increase in *VviGT14* expression and a visible reduction in *VviWRKY40* expression, whereas the expression of *VviNCED1,* which is required for ABA biosynthesis, was greatly promoted (Figure 9B). The evidence suggests that the regulation of VviWRKY40 on *VviGT14* expression is at the downstream of ABA signal transduction network. 

## 4. Discussion 

This study elucidates for the first time the involvement of VviWRKY40 in the transcriptional regulation of *VviGT14*, a key gene relating to the accumulation of glycosylated monoterpenoids in Muscat-type grape berries, using in vitro and in vivo transient expression systems. Although there are other six WRKY TFs screened out in the H1Y assay, only VviWRKY40 is confirmed to have the targeted regulatory effect on *VviGT14*. It is known that the WRKY family TFs are distributed into seven subfamilies, Ib, IIa, IIb, IIc, IId, IIIa, and IaNTWD in *Arabidopsis* [49]. VviWRKY40 is close to the AtWRKY40 in the phylogeny tree. VviWRKY40, AtWRKY18, AtWRKY40, and AtWRKY60 all belong to the WRKY IIa subfamily, AtWRKY18, AtWRKY40, and AtWRKY60 have been characterized functionally. Among these three AtWRKYs, AtWRKY18 and AtWRKY60 are transcriptional activators of ABA signaling, whereas AtWRKY40 is identified as a transcriptional repressor [50]. Our study also revealed that VviWRKY40 lacks the transcription activation function, and it represses the expression of *VviGT14*.

There have been a few studies regarding the regulation of the WRKY family TFs in grapevine. Till now, 59 WRKY proteins in grapevine have been identified [51,52]. Nevertheless, their functional research is majorly concerned about the responses to environmental stresses. For example, it has been reported that the overexpressed VviWRKY30 can enhance plant resistance to salt stress by promoting reactive oxygen species-scavenging ability and osmoticum [53]. VviWRKY8 can interact with VviMYB14, which results in the decline of resveratrol biosynthesis by downregulating the expression of *VviSTS15/21* [54]. Except these, there are no reports regarding the transcriptional regulation of VviWRKYs on the genes of the monoterpenoid biosynthetic pathway. Recently, in cotton plants, GaWRKY1 is demonstrated to activate the promoter of the sesquiterpene phytoalexin synthesis gene *CAD1-A* from transgenic *Arabidopsis* [55]. Our present study proves that VviWRKY40 is involved in regulating glycosylated monoterpenes biosynthesis. The W-box motif is generally an important binding domain of the WRKY family TFs [56]. VviWRKY40 is identified to function as a transcription repressor by binding to the w-box element in the *VviGT14* promoter region.

Abscisic acid is widely known as one of the five classic phytohormones, and it is involved in various aspects of plant growth and development. Considerable studies demonstrate that ABA is a “stress hormone” that modulates plant response to various environmental factors, including both abiotic and biotic challenges. A common phenomenon regarding the considerable accumulation of ABA is present at *véraison* of grape berry development [57]. Meanwhile, a small amount of ethylene is synthesized as well, which could induce the expression of *VviNCED*, and finally result in an increased accumulation of ABA [36]. Although many volatile compounds in grape berry are synthesized starting from the onset of ripening, the regulation of ABA on the gene expression of volatile compound biosynthetic pathway is still poorly understood. Many researches are concerned about the influence of ABA on flavonoid [58] and norisoprenoids pathway [59]. In past several years, there has been increasing evidence that plant WRKY TFs are involved in plant ABA signaling and abiotic stress responses [50,60,61]. The AtWRKY18 and AtWRKY60, together with AtWRKY40, could down regulate the expression of *AtABI5* and/or *AtABI4* genes in the ABA signaling [60]. The WRKY transcription factors operate at multiple levels in the ABA-signaling network was reported [61]. During seed germination and post-germination growth, ABA is perceived by both PYR/PYL/RCAR and ABA receptor (ABAR). AtWRKY40 could interact with ABAR, ABA perception by ABAR results in movement of the repressor protein AtWRKY40 out of the nucleus. This leads to de-repression of ABI5 at the transcriptional level. The produced ABI5 is activated following phosphorylation as a result of ABA perception by PYR/PYL/RCAR. Activation of ABI5 leads to transcription of the AtWRKY63 gene, and AtWRKY63 then activates further downstream target genes such RD29A, ABF2, and COR47. In grape berries, it has been found that among the 59 *VviWRKY*s in grapevine, 31 *VviWRKY* genes are downregulated to different extent by exogenous ABA application, whereas 14 *VviWRKY* genes are upregulated [51]. A similar effect is also observed in this study, that is, exogenous ABA application significantly reduces the *VviWRKY40* expression and elevates the *VviGT14* expression in grapevine cell suspension. It appears that the transcriptional regulation of VviWRKY40 on the *VviGT14* occurs at the downstream of ABA signaling. This also explains our observations that *VviGT14* is largely expressed and glycosylated monoterpenoids are considerably accumulated in the *véraison* stage in “Muscat blanc à Petit grain” grape berries. Certainly, the promoter region of *VviGT14* contains some ABA-responsive elements, so it is to be further determined whether the upregulation of *VviGT14* expression is as a direct consequence of ABA-downregulated *VviWRKY40* expression. 

Due to the difficulty in stable transformation in perennial plants, transient expression systems have been often used alternately to identify the function of TFs in fruit tree materials, such as apple skin [62], orange leaves [63], persimmon leaves [64], and papaya leaves [65]. In this study, we adopted grapevine cell suspension to build a dual luciferase reporter system. The effectiveness of this system has been confirmed in previous researches [40,41]. The scientists have found that the MYB family TFs can specifically activate the promoters of stilbene synthase genes in Chardonnay grapevine suspension cell cultures and a dual luciferase reporter system, and the activation effect can be reappeared in the ectopic expression of TF in grapevine hairy roots [40]. In our study, the transient gene reporter system provides a grapevine cell environment for the interaction of VviWRKY40 and the promoter of *VviGT14*, and this kind of cell environment possesses various factors that may interfere the binding. In such a grapevine suspension cell system, the suppression of VviWRKY40 on the VviGT14 promoter activity should be regarded as an actual function of the TF in grapevine. Certainly, a stable grapevine genetic transformation system should be built in future research. In addition, the concentration of glycosylated monoterpenoids is too low in the grapevine suspension cells. An accurate measurement method should be established. 

## 5. Conclusions

In conclusion, this study for the first time reports that VviWRKY40 negatively regulates the expression of *VviGT14* in ripening grape berries. This TF is involved in the regulation of glycosylated monoterpenoid biosynthesis as VviGT14 has been previously demonstrated to be a key enzyme determining the production of glycosylated monoterpenoids. Additionally, this study revealed that *VviWRKY40* expression is significantly reduced and simultaneously *VviGT14* is increased at *véraison* when glycosylated monoterpenoids rapidly accumulate. Furthermore, ABA downregulates the *VviWRKY40* expression and upregulates the *VviGT14* expression. It can be concluded that ABA promotes the *VviGT14* expression and glycosylated monoterpenoid accumulation as a result of suppressed *VviWRKY40* expression in ripening grape berries.

## Figures and Tables

**Table 1:** Putative transcription factors in DNA pull down.

Gene Id	Function
**VIT_204s0008g06000**	PREDICTED: ethylene-responsive transcription factor ERF003 [*Vitis vinifera*]
**VIT_206s0004g00490**	PREDICTED: ethylene-responsive transcription factor RAP2-11 [*Vitis vinifera*]
**VIT_209s0002g00470**	PREDICTED: ethylene-responsive transcription factor RAP2-12-like [*Vitis vinifera*]
**VIT_202s0234g00130**	PREDICTED: ethylene-responsive transcription factor2-like [*Vitis vinifera*]
**VIT_216s0013g01090**	PREDICTED: ethylene-responsive transcription factor5-like [*Vitis vinifera*]
**VIT_204s0008g05760**	PREDICTED: WRKY transcription factor 40 [*Vitis vinifera*]
**VIT_207s0129g00030**	PREDICTED: protein SHORT-ROOT-like isoform 1 [*Vitis vinifera*]
**VIT_206s0009g01790**	PREDICTED: ABSCISIC ACID-INSENSITIVE 5-like protein 2 [*Vitis vinifera*]
**VIT_205s0077g01120**	hypothetical protein VITISV_039159 [*Vitis vinifera*]
**VIT_208s0007g03900**	PREDICTED: heat stress transcription factor A-3-like [*Vitis vinifera*]

**Table 2:** Putative transcription factors in yeast one-hybrid screening analysis.

Gene Id	Function
**XM_010660100.2**	PREDICTED: probable WRKY transcription factor 22 [*Vitis vinifera*]
**XM_002272004.4**	PREDICTED: probable WRKY transcription factor 24 [*Vitis vinifera*]
**XM_002276158.4**	PREDICTED: probable WRKY transcription factor 32 [*Vitis vinifera*]
**XM_010649972.2**	PREDICTED: probable WRKY transcription factor 40 [*Vitis vinifera*]
**XM_019221865.1**	PREDICTED: probable WRKY transcription factor 26 [*Vitis vinifera*]
**XM_002272684.3**	PREDICTED: probable WRKY transcription factor 41 [*Vitis vinifera*]
**XM_010662802.2**	PREDICTED: probable WRKY transcription factor 4 [*Vitis vinifera*]

## Supplementary Materials

The following are available online at https://www.mdpi.com/2073-4425/11/5/485/s1, Figure S1: Outline of the terpenes biosynthesis in plants, Figure S2: Changes in the total souble solid content (Brix; column) and pH value (line) during the development of *Vitis vinifera* L. ‘Muscat blanc à Petit grain’ grape berries. Data are represented as means ± SD of three replicates, Figure S3: Agarose gel electrophoresis of VviWRKY40 coding sequence (left) and sodium dodecylsulfate polyacrylamide gel electrophoresis of the recombinant VviWRKY40 protein (right), Figure S4: Self-activation test of yeast one-hybrid screening, Figure S5: Agarose gel electrophoresis of the PCR products from the clones grown on the yeast-one-hybrid screening media, screening, Table S1: Primers used in this study, Table S2: Nucleotide sequence of VviGT14 fragment used in EMSA.
