# Supplementary material for: VviWRKY40, a WRKY Transcription Factor, Regulates Glycosylated Monoterpenoid Production by VviGT14 in Grape Berry
Source: Genes (Basel). 2020 Apr 29;11(5):485. doi: 10.3390/genes11050485 (PMC7290806; doi:10.3390/genes11050485)
Supplement: Supplementary file 1 [file genes-11-00485-s001.pdf]

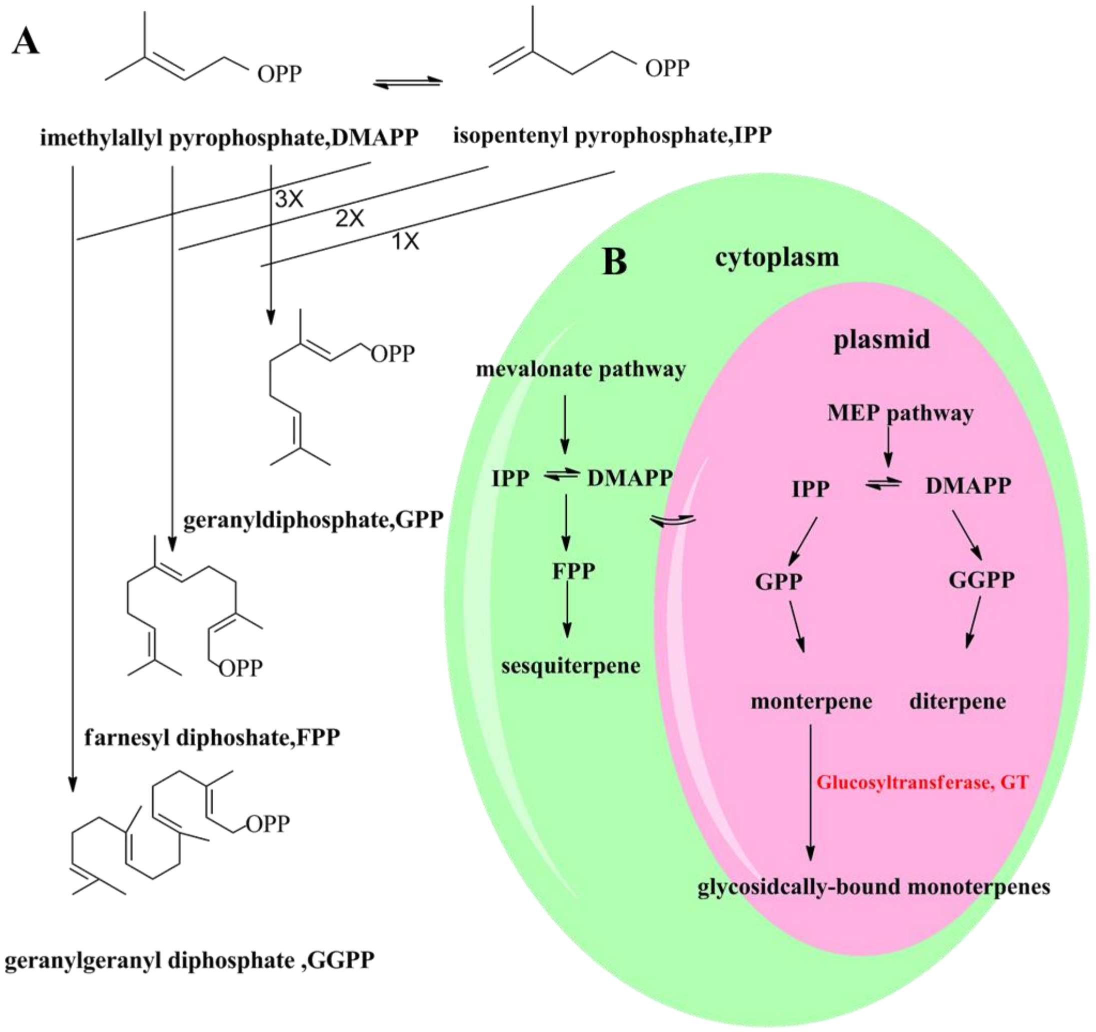

Supplemental Figure S1. Outline of the terpenes biosynthesis in plants.

A. The synthesis of allylic prenyl diphosphates dimethylallyl diphosphate (DMAPP), geranyl diphosphate (GPP) famesyl diphosphate (FPP), and geranylgeranyl diphosphate (GGPP) by583 the action of terpene synthases.

B. The schematic diagram the mevalonate and methylerythritol phosphate (MEP) pathway.

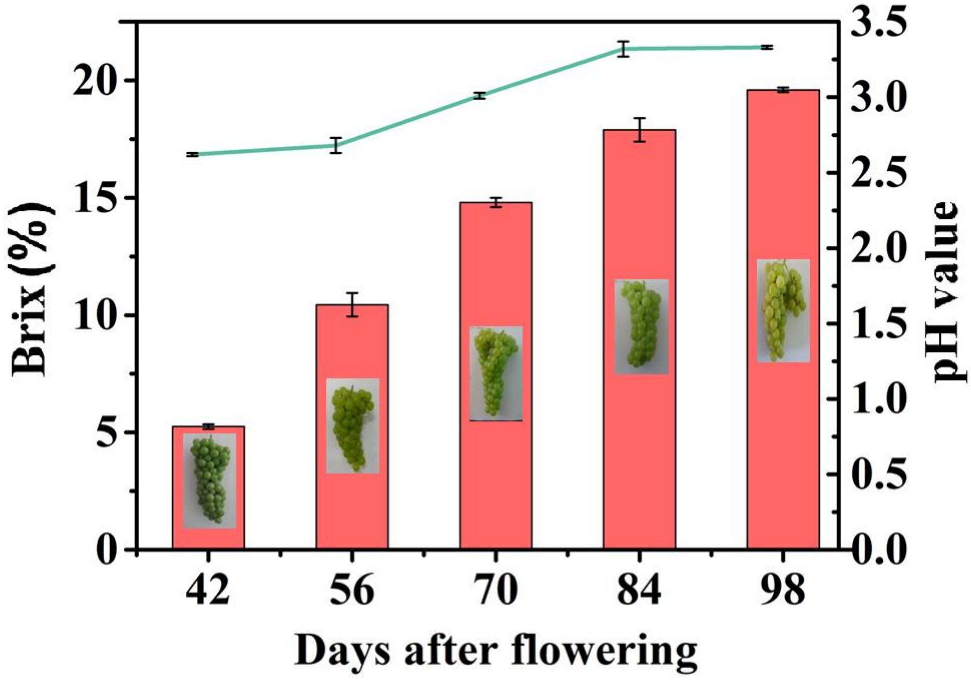

Supplemental Figure S2. Changes in the total souble solid content (°Brix; column) and pH value (line) during the development of *Vitis vinifera* L. ‘Muscat blanc à Petit grain’ grape berries. Data are represented as means ±SD of three replicates.

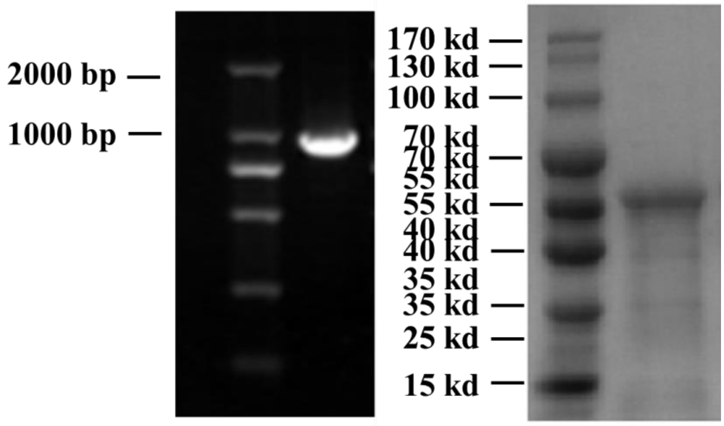

Supplemental Figure S3. Agarose gel electrophoresis of *VviWRKY40* coding sequence (left) and sodium dodecylsulfate polyacrylamide gel electrophoresis of the recombinant VviWRKY40 protein (right)

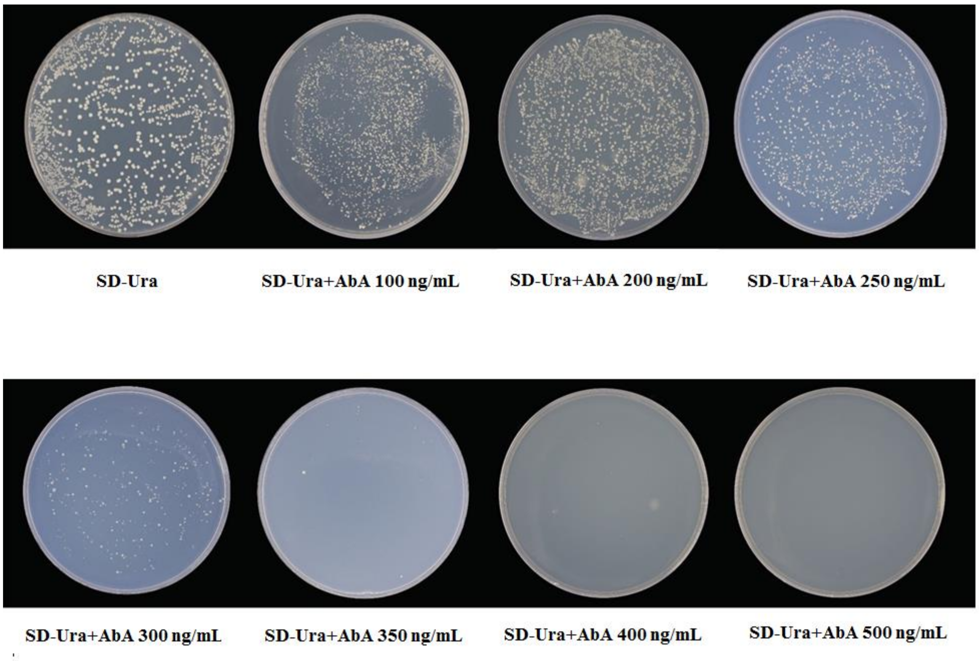

Supplemental Figure S4. Self-activation test of yeast one-hybrid screening

Yeast cells containing the bait vector pAbAi harboring the three tandem w-box elements were grown on the tryptophan SD medium without uracil (SD-Ura) supplemented with a series of concentrations of Aureobasidin A (AbA). When the concentration of AbA reaches 400 ng/mL, no any yeast grows on the medium, indicating that this AbA concentration can be used to eliminate the background and to test the validation of transcription regulators interaction with three w-box elements.

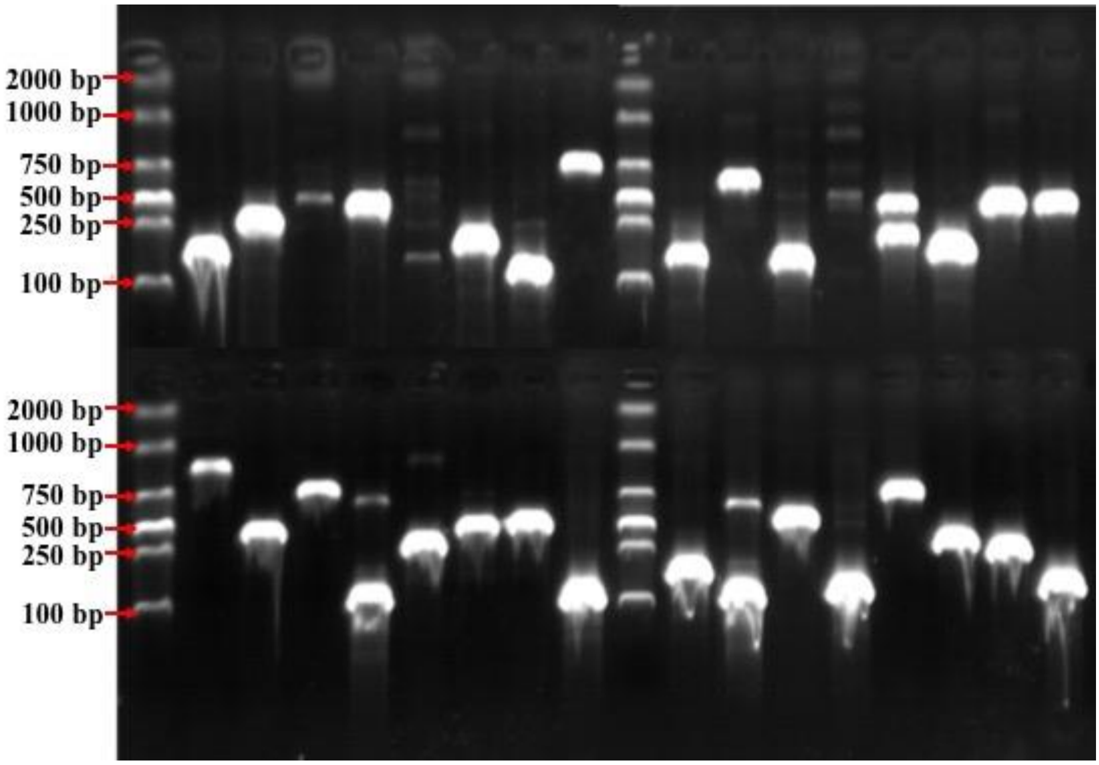

Supplemental Figure S5. Agarose gel electrophoresis of the PCR products from the clones grown on the yeast-one-hybrid screening media. The primers for PCR are M13-47 and M13-48.

Supplemental Table S1. Primers used in this study

Gene	F	R	Use in this study
**VviActin**	5'-CTTGCATCCCTCAGCACCTT-3'	5'-TCCTGTGGACAATGGATGGA-3'	Quantitative real-time PCR
**VviUbiquitin**	5'-GTGGTATTATTGAGCCATCCTT-3'	5'-AACCTCCAATCCAGTCATCTAC-3'	
**VviGAPDH**	5'-TTCTCGTTGAGGGCTATTCCA-3'	5'-CCACAGACTTCATCGGTGACA-3'	
**VviGT14**	5'-ACCATGGAGTGGAAGCATAGGG-3'	5'-TGGAAACAAGGCAGGAAAGGTG-3'	
**VviWRKY40**	5'-GCCTGCTTCTGAAGGACGGATAC-3'	5'-TGGTGAAGGTTGCTGGTGGTTATG-3'	
**VviNCED1**	5'-GCAGAGGACGAGAGTGTAAAGGA-3'	5'-GCAGAGTAAAAACACATGAAGCTAGT-3'	
**M13-47/48**	5'-CGCCAGGGTTTTCCCAGTCACGAC-3'	5'-CACACAGGAAACAGCTATGAC-3'	
**NbGT**	5'-CCGCCGTGTGATGCAGATGC-3'	5'-AGTATTGAGCTTGGCGAGCAACTC-3'	
**NbEF1α**	5'-AGCTTTACCTCCCAAGTCATC-3'	5'-AGAACGCCTGTCAATCTTGG-3'	
**VviWRKY4**	5'-ATGGCTCAGAATGATACGTCCTCG-3'	5'-TTACACCGCAATCTGCTCTT-3'	Cloning of full-length gene
**VviWRKY22**	5'-ATGATGAGCGAGTTTTTGGGC-3'	5'-TCAGCAACCGCCGGCGGCAGTT-3'	
**VviWRKY24**	5'-ATGGGTTCTTCCTCTGGGAGC-3'	5'-CCGGGACAGAGCAGAGACTCGAATAACATG-3'	
**VviWRKY26**	5'-ATGGCTTCCTCTGCTGCTAGTTTT-3'	5'-TGGTTCTTGAGAAAACCCCATCTGGG-3'	
**VviWRKY32**	5'-ATGGCTGGGAACCAGAGCT-3'	5'-ACGGCTTGATTTCAAATCCA-3'	
**VviWRKY41**	5'-ATGGACACTGGTTTGAAATGGCA-3'	5'-CTAGGAGAAAAATCCTGGGGTAT-3'	

Supplemental Table S2. Nucleotide sequence of *VviGT14* fragment used in EMSA

Name	Sequence
Fragment	AGAAGTGAGAGGGTAAG**TTGAC**CATGCACTCTCATCTAGAT

Note：w-box in the fragment is highlighted in yellow
